# Surrogates for Liquid–Liquid Extraction

**DOI:** 10.1021/acsomega.3c08140

**Published:** 2023-12-14

**Authors:** Maximilian Neubauer, Georg Lenk, Nikolai Josef Schubert, Susanne Lux, Thomas Wallek

**Affiliations:** † Institute of Chemical Engineering and Environmental Technology, Graz University of Technology, Inffeldgasse 25C, 8010 Graz, Austria; ‡ OMV Downstream GmbH, Trabrennstraße 6-8, 1020 Vienna, Austria

## Abstract

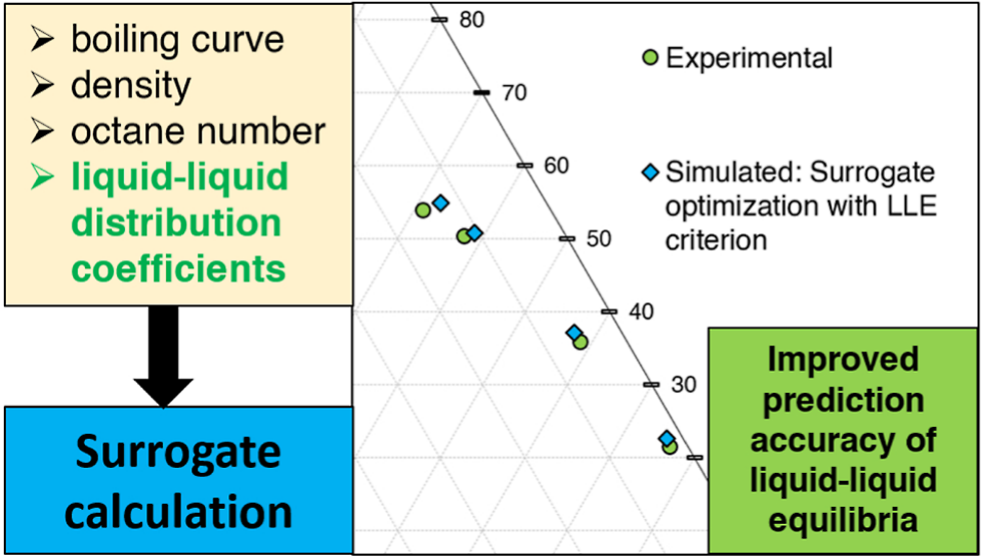

Fuel surrogates are mixtures that mimic the properties of real fuels with only a small number of components, simplifying the calculation and simulation of fuel-related processes. This work extends a previously published surrogate optimization algorithm toward the generation of fuel surrogates with a focus on liquid–liquid extraction characteristics. For this purpose, experimental liquid–liquid equilibrium data from batch extraction experiments are incorporated into the calculation procedure as an additional constraint. The use of the method is demonstrated by optimizing a surrogate for the catalytic reformate. Application of the surrogate to an extraction process and comparison with experimental data demonstrate that the resulting surrogate accurately depicts the properties of the real mixture with regard to liquid–liquid extraction performance. This demonstrates that the use of such surrogates is of particular interest for mixtures used as extracting agents for biofuels.

## 1 Introduction

Decarbonization of the transport sector remains one of the biggest challenges to reaching the net-zero carbon neutrality target for 2050. In this context, biofuels are an important contribution toward achieving that goal. They provide a low-net-emission energy source for light-duty vehicles in the short term and heavy-duty vehicles such as trucks, aircraft, and ships in the medium to long term.^1,2^ In the sector of liquid biofuels, ethanol and biodiesel had the largest market share (66 and 28%, respectively) in 2022.^3^ Other biofuel types, like biomethanol,^4^ biopropanol,^5^ biobutanol,^6^ or derived compounds,^7^ are currently under development.

Regardless of which biofuels are in use now or will be in the future, developing energy-efficient production processes is crucial from both an economic and environmental point of view. For the production of bioalcohols, workup from aqueous solutions is commonly the case. The formation of azeotropes with water (for ethanol, propanol, and butanol) makes azeotrope separation processes necessary in order to obtain purified components. Most commonly, extraction, extractive distillation, or (heterogeneous) azeotropic distillation are employed. What they all have in common is that a solvent must be used that must be regenerated, resulting in a high energy demand. If the biofuels are to be used in combination with hydrocarbon fuels, acting as a substitute of a certain percentage, as for instance outlined in the Renewable Energy Directive of the European Union,^8^ a promising process alternative is the extraction of the biofuel components directly into the hydrocarbon fuels (e.g., gasoline)^9,10^ or blending stocks (e.g., catalytic reformate).^11^ This omits the step of solvent recovery, and only relatively small amounts of water in the organic extract phase need to be removed in order to meet the final fuel specifications.

In the context of fuel research, process simulation has gained immense traction in the last few years. Validated with experimental data in the optimal case, process simulations can help to achieve significant cuts in process development time and, thus, costs. Sound physical property data and thermodynamic models are key to achieving reliable and accurate results. Transforming a static process model, which is often the starting point in early stage development, into a fully dynamic model with good reliability and accuracy (i.e., a digital twin) requires rigorous thermodynamics in order to be able to depict changes with varying input. With the increasing complexity of the systems at hand and the increasing number of components, applying rigorous thermodynamics can be challenging. The direct workup of biofuels with gasoline or blending stocks, such as reformate, as mentioned in the paragraph above, represents such a case.

Gasoline and blending stocks, such as reformate, are complex hydrocarbon mixtures consisting of hundreds of components. A common way to model and simulate such systems is the pseudocomponent approach, which has been in use for several decades now. Although it is still widely utilized, several limitations for pseudocomponents in general can be identified:^12^

- In certain processes, the chemical character of a mixture may be important to consider in terms of chemical reactions occurring. However, for pseudocomponents, no chemical character can be defined.
- The definition of pseudocomponents is primarily based on (pseudo)-boiling points and some other parameters such as molar mass, viscosity, or specific gravity. If other physical properties are needed for simulation, they have to be estimated, with the reliability of established estimation methods for the acentric factor or critical properties often being limited.
- Group contribution methods like Universal Quasichemical Functional Group Activity Coefficients (UNIFAC) for the estimation of binary interaction parameters cannot be used with pseudocomponents due to the unavailability of a molecular structure.
- Commercial simulation programs do not support arbitrary combinations of real components in the original mixture and pseudocomponents. This means that real components from the original mixture cannot be placed in the middle of the boiling range used for the definition of the pseudocomponents without knowledge about the actual composition.

One could argue that in the age of ever-increasing computing power, the number of components in simulations does not matter anymore. However, there are several reasons to keep the number of components reasonably low. First of all, the dimension of unit operation models depends on the number of components, which can lead to problems, especially when equation-oriented simulators are used due to internal limits and memory requirements.^12^ Second, rigorous thermodynamics are stretched to their limits in the case of thermodynamic equilibrium calculations (liquid–liquid, vapor–liquid), involving systems containing several hundred components. On the one hand, qualitative and quantitative analysis of each single component in these mixtures is a tedious and time-consuming task. On the other hand, even if the exact composition is known, physical property data for all pure components as well as interaction parameters for different thermodynamic models have to be gathered and validated. For instance, considering a fuel consisting of *n* = 200 real components results in 19,900 binary subsystems. In the case of the nonrandom two-liquid (NRTL) model, this requires 19,900  binary parameter sets to be validated. Even if group contribution methods such as UNIFAC are used, the predictions must be compared to experimental data for validation.

Hence, it becomes obvious that for efficient and flexible modeling of real fuels, a reduction in the number of components is desirable. For this purpose, the generation of surrogates, representing a mixture with a limited number of components that mimics the properties of the real fuel, has become a frequently used approach in the area of combustion engine research.^13−15^ Here, combustion and emission simulations play an important role. In the course of surrogate generation, the composition of the surrogate is adjusted in such a way that it represents the key properties of the real fuel. Examples of such key properties are standardized ratings like distillation characteristics, octane number, and density, or nonstandardized values like the C/H ratio, viscosity, and heating value. Once the target properties are defined, the composition of the surrogate is calculated by an algorithm which is designed to minimize an objective function that accounts for deviations between the given properties of the real fuel and the calculated properties of the surrogate.^16^

To the best of the authors’ knowledge, incorporating the extractive characteristics of real fuels with respect to liquid–liquid equilibria (LLE) into surrogates has not been attempted yet. Particularly in view of the potential of hydrocarbon fuels as workup solvents in biofuel production and the immense benefit of digital twins in process development and operation, the generation of such surrogates is of considerable interest. Consequently, the goal of this paper is to develop surrogates that mimic the extractive properties of real hydrocarbon fuels, focusing on the extraction of 1-propanol from a aqueous solution with a reformate as a solvent. The benefit of the resulting surrogates is demonstrated by their practical application in a multistage extraction process.

The paper is structured as follows. In Section 2, the experimental determination of LLE tie lines is presented, along with other data required as input to the algorithm. Section 3 outlines the surrogate optimization algorithm used, focusing on its extension by an LLE criterion to incorporate the surrogates’ extractive characteristics. Section 4 presents and discusses the results, including experimental tie lines, the resulting surrogate mixture for liquid–liquid extraction, and finally, its application for an exemplary multistage extraction process. Section 5 provides a summary of the work and a conclusion about the results.

## 2 Experimental Section

For the optimization of the surrogates in view of extractive performance, experimental tie line data for the ternary system 1-propanol–water–reformate was collected. This data was then used as an input for the algorithm.

**Figure 1:**
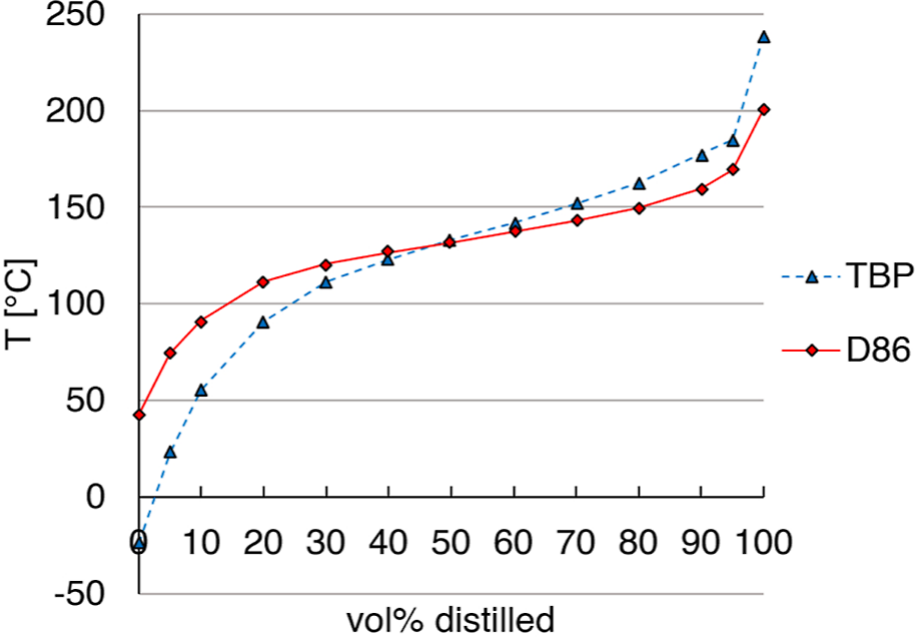
ASTM D86 and TBP curves of reformate, TBP curve calculated according to Daubert^17^ from ASTM D86 data provided by OMV Downstream GmbH.

1-Propanol (≥99.5%) and double-distilled water (conductivity ≤ 0.02 μS/cm) were obtained from Carl Roth GmbH. Reformate was provided by OMV Downstream GmbH. The reformate properties are listed in Table 1. The ASTM D86 and true boiling point (TBP) distillation curves of reformate are shown in Figure 1.

**Table 1:** Key Properties of Reformate as Provided by OMV Downstream GmbH

property	value	unit
density at 15 °C	828	kg/m^3^
research octane number (RON)	101.8	-
molar mass	103.0	g/mol
aromatics content	75.4	vol %

In order to generate experimental tie lines, a certain amount of 1-propanol was added to a mixture of water and reformate (50:50 wt %) in a tempered 200 mL separation funnel at 23 °C. The mixture was agitated in a mechanical shaking rack at 200 rpm for 2 h. The extract and raffinate phases were separated after overnight settling. The 1-propanol content in both phases and the reformate content in the raffinate phase were analyzed by gas chromatography (Agilent 6890N) with a flame ionization detector. Details concerning the gas chromatography method can be found in the Supporting Information. The water content in the extract phase was determined by Karl Fischer titration (SI Analytics TITRONIC 500). The reformate content in the extract and the water content in the raffinate were calculated via mass balances.

## 3 Algorithm for Surrogate Optimization

The algorithm used for the calculation of the surrogate composition was initially developed by Reiter et al. for the generation of surrogates for crude oil, diesel, biodiesel, and mixtures thereof.^18−20^ A subsequent adaptation by Grubinger et al. focused on gasoline surrogates.^16^ Preliminary investigations revealed that surrogates with a focus on boiling curve, density, octane number, and molar mass do not depict the extractive properties in the context of liquid–liquid equilibria with sufficient accuracy, which will be illustrated in Subsection 4.2. To overcome this, experimental liquid–liquid equilibrium data in the form of distribution coefficients had to be implemented as an additional criterion for the optimization procedure. This extension will be outlined in Subsection 3.2.5 in detail.

### 3.1 Selection of Components

The original database established by Grubinger et al.^16^ for gasoline, comprising 30 components, was extended to 40 components. Since reformate is rich in aromatics (75.4 vol %), 10 additional aromatic components were considered, 5 to supplement the medium-boiling region and 5 to supplement the high-boiling region, the latter to improve the steep curvature on the right-hand side of the boiling curve, cf. Figure 1. The final components used for surrogate generation are listed in Table 2.

**Table 2:** Component List for Surrogate Calculation, Including Molar Mass, *M*, Mass Density, ρ, Research Octane Number, RON, and Normal Boiling Point, *T*_boil_

CAS number	component	*M* [g/mol]	ρ [g/cm^3^]	RON [—]	*T*_boil_ [°C]
106-97-8	butane	58.1222	0.579	93.8	–0.66
463-82-1	neopentane	72.1488	0.591	85.5	9.36
78-78-4	isopentane	72.151	0.6234	92.3	27.84
109-66-0	pentane	72.1488	0.6214	61.8	36.21
75-83-2	neohexane	86.178	0.6445	91.8	49.74
107-83-5	isohexane	86.178	0.6485	73.4	60.23
96-14-0	3-methylpentane	86.178	0.6598	74.5	63.24
110-54-3	hexane	86.1754	0.6627	24.8	68.81
71-43-2	benzene	78.1118	0.8737	99	79.9
591-76-4	isoheptane	100.202	0.6787	42.4	90.37
142-82-5	heptane	100.202	0.6868	0	98.39
540-84-1	isooctane	114.229	0.695	100	99.03
108-87-2	methylcyclohexane	98.189	0.7724	74.8	101.25
108-88-3	toluene	92.141	0.87	112	110.62
111-65-9	octane	114.229	0.7054	–19	125.57
1678-91-7	ethylcyclohexane	112.213	0.79068	45.6	131.79
100-41-4	ethylbenzene	106.168	0.87	107	136.5
106-42-3	*p*-xylene	106.168	0.8642	127	138.35
108-38-3	*m*-xylene	106.16	0.8669	124	138.85
95-47-6	*o*-xylene	106.168	0.8831	103	144.7
111-84-2	nonane	128.255	0.7202	–17	150.82
98-82-8	isopropylbenzene	120.194	0.846753	105.1	153.05
1678-92-8	propylcyclohexane	126.239	0.796	17.8	156.72
103-65-1	propylbenzene	120.192	0.8648	129	159.22
108-67-8	mesitylene	120.192	0.8652	137	164.72
98-06-6	2-methyl-2-phenylpropane	134.221	0.841327	116.1	168.59
95-63-6	1,2,4-trimethylbenzene	120.192	0.8797	110.42	169.36
538-93-2	isobutylbenzene	134.221	0.81517	102.8	172.78
135-98-8	2-phenylbutane	134.221	0.85192	102.8	175.3
526-73-8	1,2,3-trimethylbenzene	120.194	0.887138	98.8	176.08
1678-93-9	butylcyclohexane	140.266	0.80145	–8.1	180.89
104-51-8	butylbenzene	134.221	0.8648	104.41	183.3
493-02-7	*trans*-decalin	138.25	0.8733	39.78	186.98
493-01-6	*cis*-decalin	138.25	0.9012	39.78	195.75
119-64-2	tetralin	132.205	0.9739	100.25	207.2
1077-16-3	hexylbenzene	162.275	0.828263	56.4	226.1
90-12-0	1-methylnaphthalene	142.2	0.988603	108	244.61
1078-71-3	heptylbenzene	176.302	0.817845	27.7	246
92-52-4	phenylbenzene	154.211	0.996942	158	255.05
2189-60-8	octylbenzene	190.329	0.814158	–0.6	264.5

### 3.2 Selection and Modeling of Target Properties

The target properties chosen for the optimization procedure include the TBP distillation curve, density, research octane number, molar mass, and aromatic fraction. Additionally, the distribution coefficients of 1-propanol and water between extract and raffinate were added as critera for modeling the liquid–liquid extraction behavior.

#### 3.2.1 Boiling Curve

A stepwise approximation method presented by Reiter et al.^19^ was used to model the TBP curve; for details, we referred to their work. The available distillation curve data for the reformate, cf. Figure 1, was generated according to the ASTM D86 standard. However, as the above-mentioned approximation method does not work with D86 curves as input, it had to be converted into a TBP curve first, which was done according to the method proposed by Daubert.^17^ Since D86 curves are based on volume fractions, φ_*i*_, the obtained TBP curves obtained through conversion are also volume-based. Due to the fact that parts of the objective function of the optimization algorithm are based on mass fractions, *w*_*i*_, eq 1 was used for conversion, where *v*_NBP_ represents the molar volume at normal boiling point, and *M*_*i*_ is the molar mass for each component.
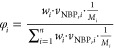
1

**Figure 2:**
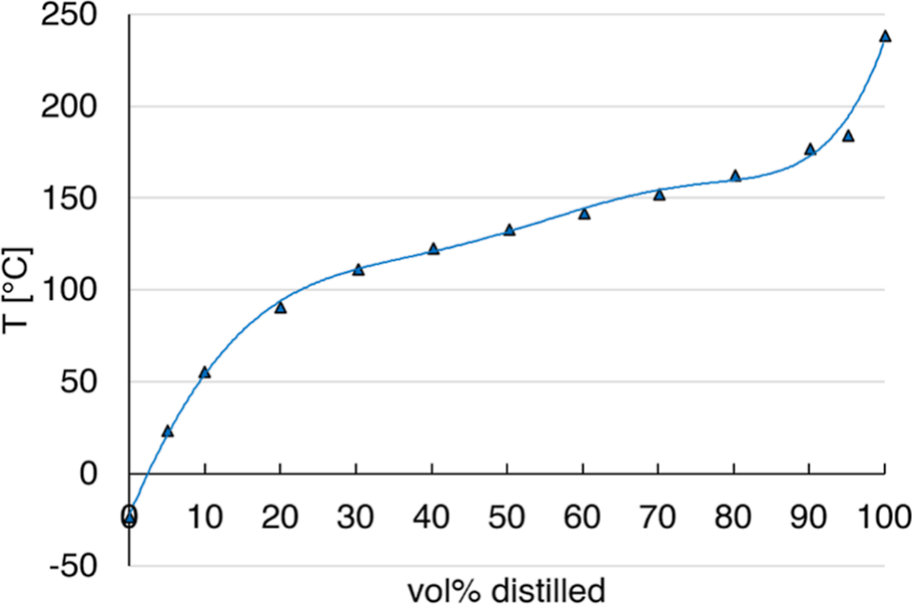
Sixth order polynomial fit for the TBP curve.

The TBP data was approximated by least-squares fitting based on a sixth-order polynomial, in order to provide an algebraic input function for the optimization algorithm. This is shown in Figure 2.

#### 3.2.2 Density

The liquid density of the mixture at 15 °C was calculated, as shown in eq 2.
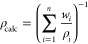
2

In this ideal mixing model, *w*_*i*_ stands for the mass fraction of the component, *i*, and ρ_*i*_ stands for its liquid density at 15 °C. According to Reiter et al.^19^ and several other works,^21−23^ the ideal linear mixing rule can be applied with satisfactory results to diesel and gasoline.

#### 3.2.3 Research Octane Number

Like the liquid density of the mixture, the RON is also calculated by a linear mixing model using volume fractions, φ_*i*_, according to eq 3.
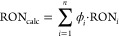
3

Despite its simplicity, the model provided satisfactory results for predicting the cetane number of diesel^19,20^ and kerosene surrogates.^23^ RON numbers for the starting basis of 30 components were taken from Grubinger et al.,^16^ while the RON numbers for the additional 10 aromatic components were estimated by the method proposed by Albahri.^24^

#### 3.2.4 Molar Mass

The molar mass is calculated from the molar masses of the individual components, *M*_*i*_, and the molar fractions, *x*_*i*_, according to eq 4.
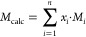
4

#### 3.2.5 Distribution Coefficients

**Figure 3:**
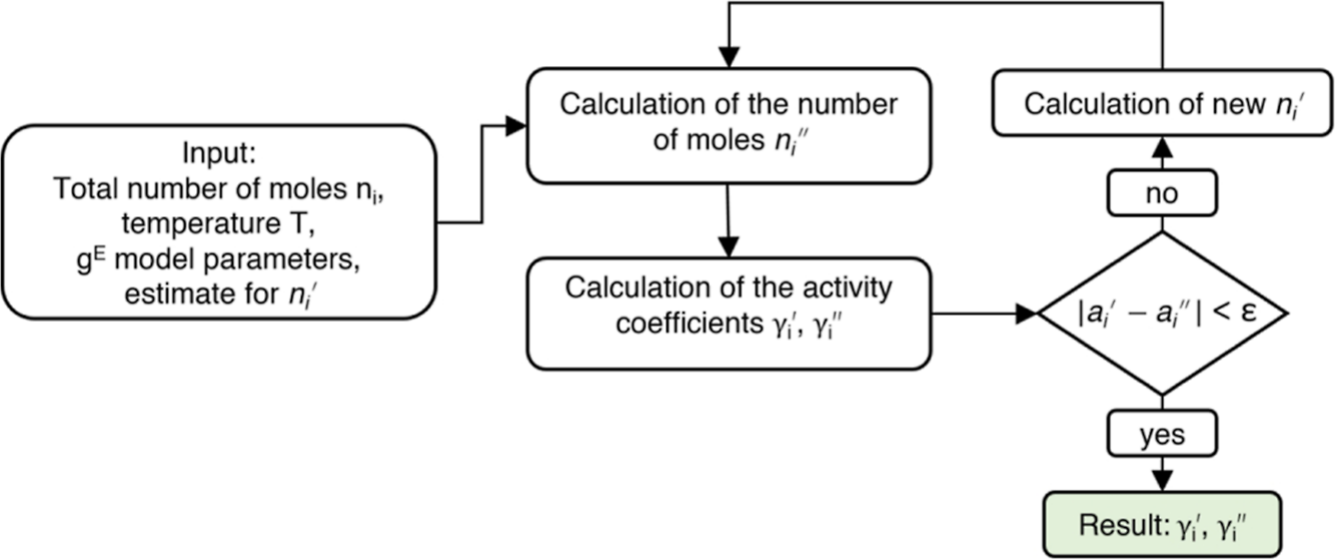
LLE flash calculation flowsheet, adapted from Gmehling et al.^25^ Page 274. 2019. Copyright 2019 Wiley-VCH GmbH. Reproduced with permission.

The calculated distribution coefficients of 1-propanol and water between the extract and raffinate phases are obtained from the results of a LLE flash calculation using the *K*-factor method, as described by Gmehling et al.^25^ It is graphically depicted in Figure 3.

First, an estimate is made for the mole number *n*_*i*_^′^ in the first liquid phase. From the total number of moles *n*_*i*_, the mole numbers *n*_*i*_^″^ in the second liquid phase are calculated. The next step is the determination of the activity coefficients of the components in both phases, γ_*i*_^′^ and γ_*i*_^″^, by the chosen activity coefficient model. Then, the fulfillment of the isoactivity condition within a numerical threshold, ε, is checked, which will, expectedly, not be the case after the first iteration. For consecutive iterations, new mole number estimates *n*_*i*_^′^ for the first liquid phase must be generated. This is done by eq 5, the derivation of which can be found in Gmehling et al.^25^
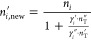
5where *n*_T_ designates the total number of moles in each phase. If the difference in activities is below the given threshold, ε, then the iteration is stopped. For the calculation of the activity coefficients, UNIFAC-liquid–liquid (UNIFAC-LL)^26^ and NRTL^27^ were used. Concerning the NRTL model, 89% of the  binary interaction parameters were not available for the 40 components listed in Table 2. These were estimated with the AspenPlus (V11) process simulation software using different variants of the UNIFAC family of group contribution methods, in particular, standard UNIFAC,^28^ UNIFAC-LL^26^ and UNIFAC-Dortmund (UNIFAC-DMD).^29^ Concerning standard UNIFAC and UNIFAC-DMD, revised and extended parameters from the UNIFAC Consortium^30^ (2021 edition) as well as default parameters from AspenPlus were used.

### 3.3 Determining the Surrogate Composition

The basic algorithm utilized for determining the surrogate composition was published by Reiter et al. for crude oil, fossil diesel, biodiesel, and mixtures thereof.^18−20^ For the present paper, the original Fortran 2008 code was adapted and translated into the Wolfram Language.^31^ The core of the algorithm is an objective function to be minimized, with the structure being as follows
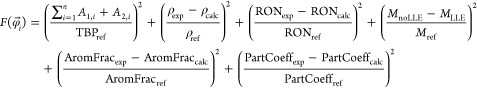
6

All properties considered in the objective function depend on the composition of the surrogate in terms of the vector of volume fractions, , or other concentration measures derived from . The first fitting term describes the fit of the TBP curve, where two possible partial contributions *A*_1,*i*_ and *A*_2,*i*_ are summed for all components. The sum of these partial contributions corresponds to an average deviation of the TBP curve of the surrogate from that of the target fuel, which is explained in more detail in previous literature.^19^ The second fitting term is the density criterion. The third fitting term is the research octane number (RON) criterion. The fourth fitting term is the averaged molar mass of reformate. The fifth fitting term is the aromatics content. The sixth fitting term is the extraction criterion to mimic a separation funnel experiment. The denominators of the objective function, indexed with “*ref*”, correspond to weighting factors. Their numerical values are listed in the Supporting Information.

## 4 Results and Discussion

### 4.1 Experimental Results

The experimentally determined LLE data are shown in Table 3.

**Table 3:** Experimentally Determined LLE Data (Weight Fractions) for System 1-Propanol (1), Water (2), and Reformate (3) at *T* = 23 °C and *P* = 1 atm^a^

extract (organic) phase		raffinate (aqueous) phase	
*w*_1_ propanol	*w*_2_ water	*w*_1_ propanol	*w*_2_ water
0	0.0002	0	0.9999
0.113	0.0080	0.156	0.8435
0.215	0.0211	0.174	0.8251
0.286	0.0374	0.187	0.8119
0.358	0.0547	0.195	0.8035
0.406	0.0733	0.203	0.7959
0.481	0.1055	0.215	0.7836
0.502	0.1200	0.211	0.7867
0.517	0.1343	0.220	0.7780
0.538	0.1508	0.225	0.7728

a Standard deviations: σ(*w*_Propanol_) = 0.003, σ(*w*_Water_) = 0.0002.

### 4.2 Surrogate without Liquid–Liquid Criterion

**Figure 4:**
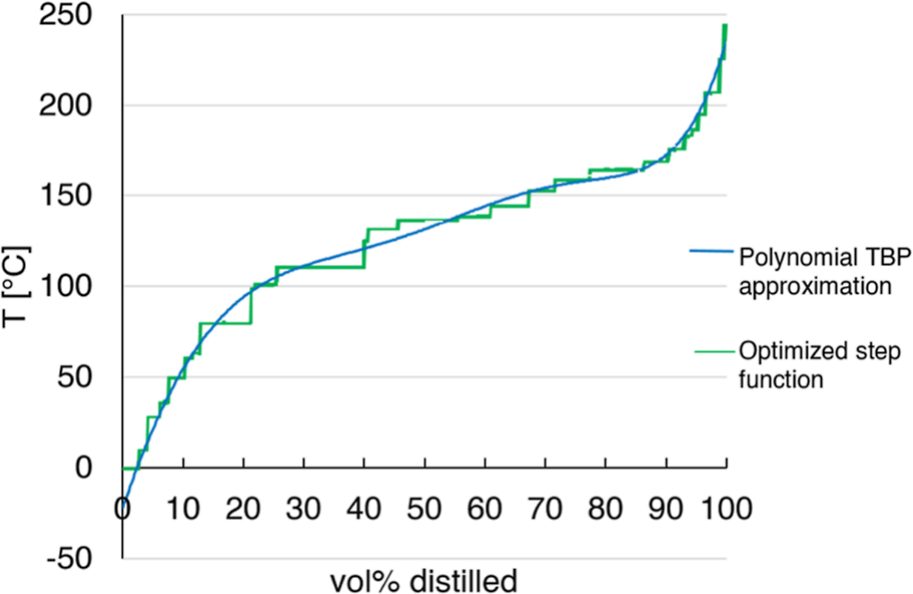
TBP boiling curve of the resulting surrogate without the LLE criterion.

First of all, a surrogate mixture without the LLE criterion, i.e., neglecting the last term of the objective function (eq 6), was calculated to assess its suitability for LLE calculations. From the 40 components listed in Table 2, the surrogate optimization algorithm selected 29 to be significant. This composition is given in the Supporting Information. The TBP boiling curve of the resulting surrogate is shown in Figure 4, and its deviations from the target values are listed in Table 4.

**Table 4:** Deviations from Target Values of Conventional Surrogate

target criterion	unit	experimental	calculated	relative error [%]
ρ	kg/m^3^	0.828	0.8279	–0.012
RON	-	101.8	101.799	–0.001
molar mass	g/mol	103.0	102.393	–0.589
aromatics fraction	-	0.754	0.7538	–0.024

**Figure 5:**
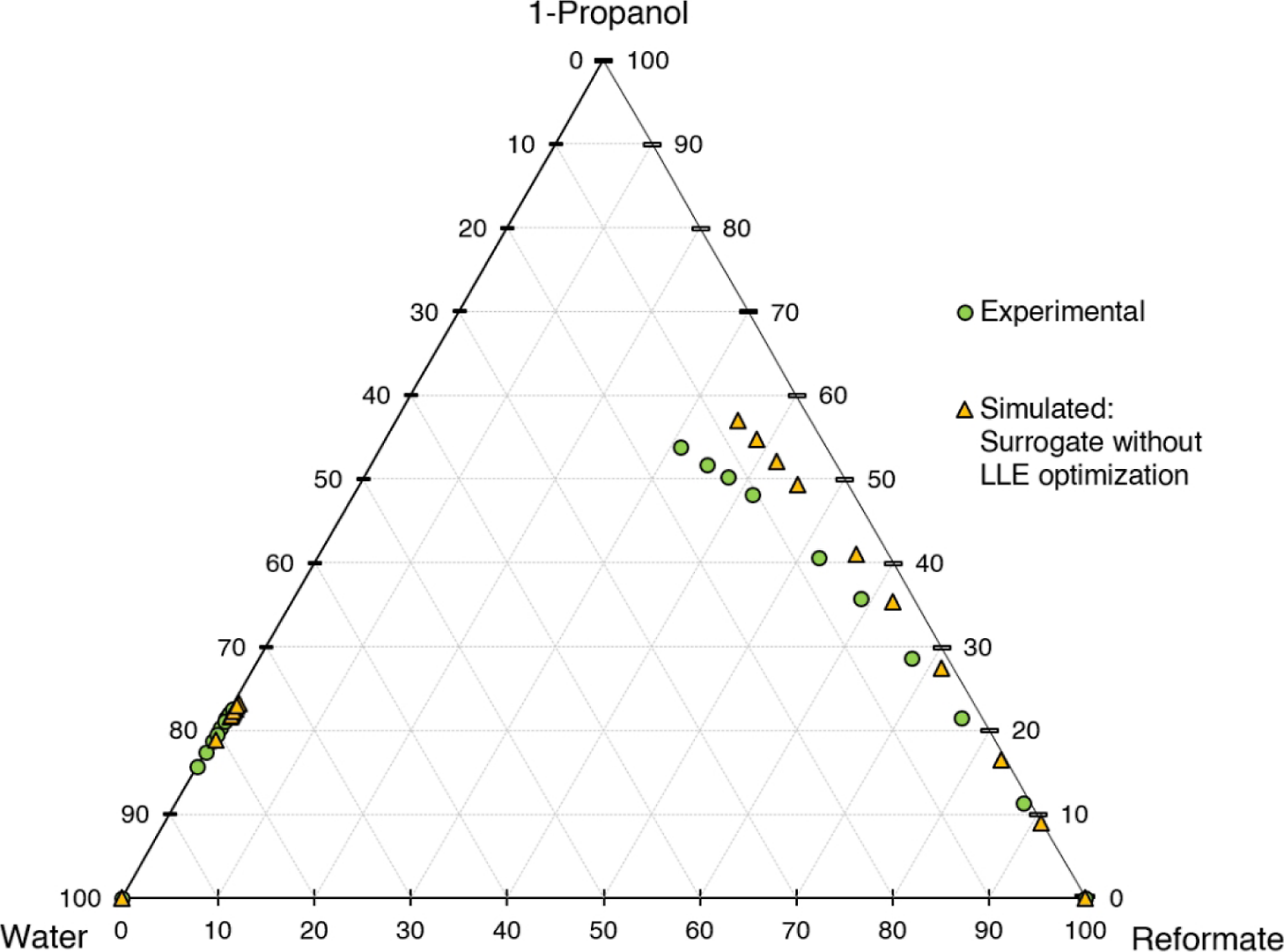
Ternary diagram of system 1-propanol–water–reformate: comparison between experimental LLE data and simulated results with conventional surrogate without LLE criterion, simulation conducted in AspenPlus V11 with NRTL-UNIFAC21.

Although this surrogate excellently replicates the boiling curve and other key properties, this is not the case for LLE. As shown in Figure 5, the surrogate does not depict the mixing gap with satisfying accuracy. The mixing gap predicted by NRTL-UNIFAC21 is significantly larger than that experimentally determined, thus underestimating the water content in the extract. Higher water contents in the extract result in increased separation demand for extract refining; therefore, the conventional surrogate would clearly underestimate the effort needed for refinement of the extract phase. This indicates the necessity of including the LLE criterion for the surrogate calculation by considering the liquid–liquid distribution coefficients to depict the mixing gap correctly.

### 4.3 Surrogate Including the Liquid–Liquid Criterion

For the surrogate generation, the LLE data were split into two parts. Table 5 shows the data points used for calibration, while the data points used for validation of the surrogate are shown in Table 6.

**Table 5:** LLE Data Points Used for Calibration^a^

extract (organic) phase		raffinate (aqueous) phase	
*w*_1_ propanol	*w*_2_ water	*w*_1_ propanol	*w*_2_ water
0.113	0.0080	0.156	0.8435
0.286	0.0374	0.187	0.8119
0.406	0.0733	0.203	0.7959
0.481	0.1055	0.215	0.7836
0.517	0.1343	0.220	0.7780

a Standard deviations: σ(*w*_Propanol_) = 0.003, σ(*w*_Water_) = 0.0002.

**Table 6:** LLE Data Points Used for Validation^a^

extract (organic) phase		raffinate (aqueous) phase	
*w*_1_ propanol	*w*_2_ water	*w*_1_ propanol	*w*_2_ water
0.215	0.0211	0.174	0.8251
0.358	0.0547	0.195	0.8035
0.502	0.1200	0.211	0.7867
0.538	0.1508	0.225	0.7728

a Standard deviations: σ(*w*_Propanol_) = 0.003, σ(*w*_Water_) = 0.0002.

**Figure 6:**
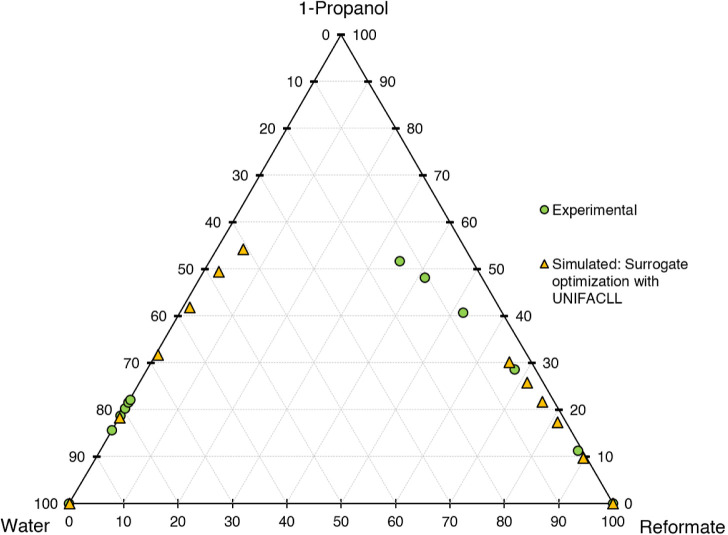
Ternary diagram of system 1-propanol–water–reformate, comparison between experimental LLE data and simulated results with LLE-optimized surrogate for the calibration set, simulation conducted in AspenPlus V11 with UNIFAC-LL.

**Figure 7:**
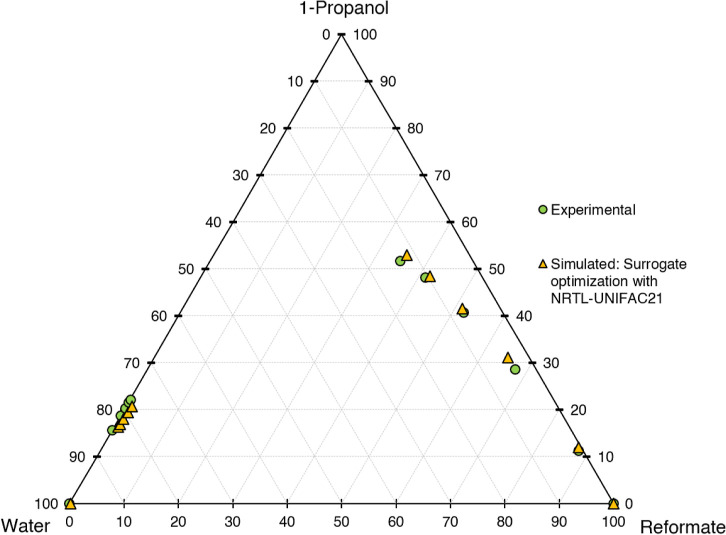
Ternary diagram of system 1-propanol–water–reformate, comparison between experimental LLE data and simulated results with LLE-optimized surrogate for the calibration set, simulation conducted in AspenPlus V11 with NRTL-UNIFAC21.

**Figure 8:**
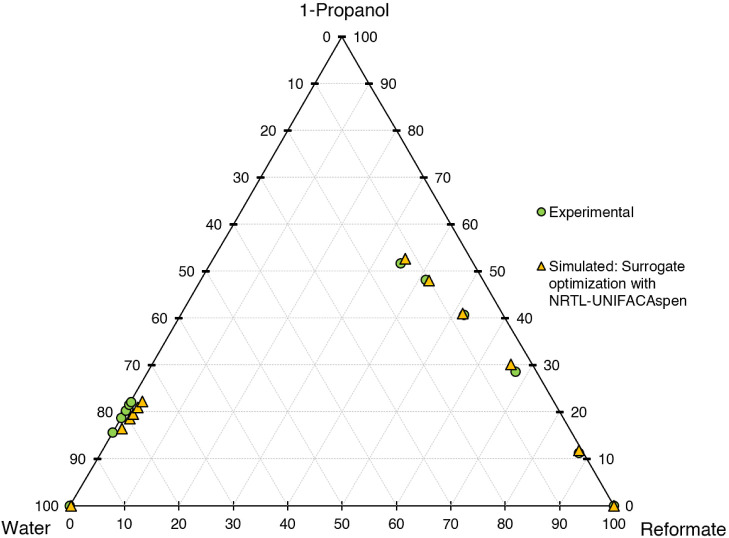
Ternary diagram of system 1-propanol–water–reformate, comparison between experimental LLE data and simulated results with LLE-optimized surrogate for the calibration set, simulation conducted in AspenPlus V11 with NRTL-UNIFACAspen.

**Figure 9:**
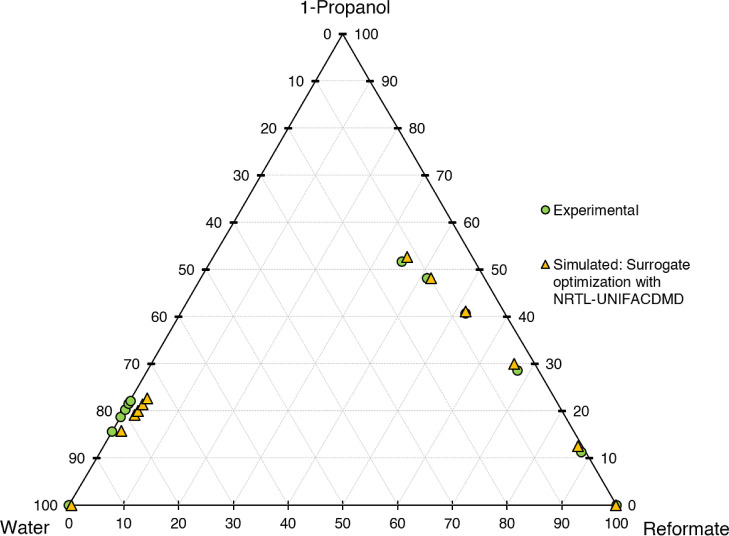
Ternary diagram of system 1-propanol–water–reformate, comparison between experimental LLE data and simulated results with LLE-optimized surrogate for the calibration set, simulation conducted in AspenPlus V11 with NRTL-UNIFACDMD21.

**Figure 10:**
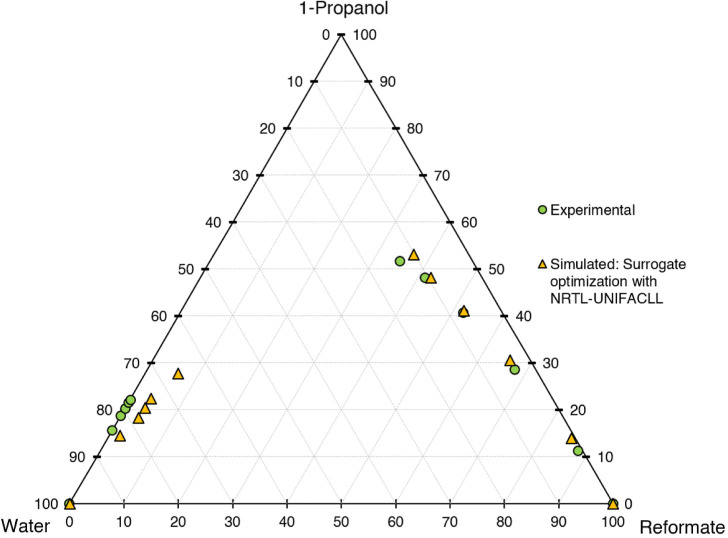
Ternary diagram of system 1-propanol–water–reformate, comparison between experimental LLE data and simulated results with LLE-optimized surrogate for the calibration set, simulation conducted in AspenPlus V11 with NRTL-UNIFACLL.

As explained in the Subsection 3.2.5., different activity coefficient models were used for the LLE flash calculation, which is part of the surrogate optimization procedure. The results for the different models are shown in Figures 6–10. The calculation time for the surrogate calculation ranged between 13 and 125 h, depending on the activity coefficient model used, compared to 2 min without LLE optimization.

First, the different models shall be evaluated qualitatively on a graphical basis in ternary diagrams, comparing the calibration results with experimental data. UNIFAC-LL does not yield satisfactory results, with the slope of the tie lines (=selectivity) being depicted wrongly. Better results are achieved for the NRTL model with different UNIFAC variants employed for the estimation of missing parameters. The results are quite similar, with the major difference being the depiction of the raffinate phase, with NRTL-UNIFAC21 being closest to the experimental values and NRTL-UNIFACLL having the largest deviations. Considering both the raffinate and extract phases, NRTL-UNIFAC21 yields the best compromise in depicting 1-propanol, water, and reformate content with satisfying accuracy and is thus chosen for further investigations. For new systems, e.g., different fuel compositions and/or different alcohols, it is recommended to repeat the model comparison for reliable results, since certain systems are depicted better by some models than others. The final composition of the LLE-optimized surrogate is shown in Table 7, and the deviations from the target values are shown in Table 8.

**Table 7:** Composition of the LLE-Optimized Surrogate with NRTL-UNIFAC21 (Target Values: TBP, Density, RON, Aromatics Fraction, Molar Mass, and Liquid–Liquid Distribution Coefficients)

component	*w* [%]
butane	15.46
benzene	11.06
toluene	28.02
tetralin	45.45

**Table 8:** Deviations from Target Values of LLE-Optimized Surrogate

target criterion	unit	experimental	calculated	relative error [%]
ρ	kg/m^3^	0.828	0.846	2.135
RON	-	101.8	102.083	0.278
molar mass	kg/kmol	103.0	94.734	–8.025
aromatics fraction	-	0.754	0.803	6.561

**Figure 11:**
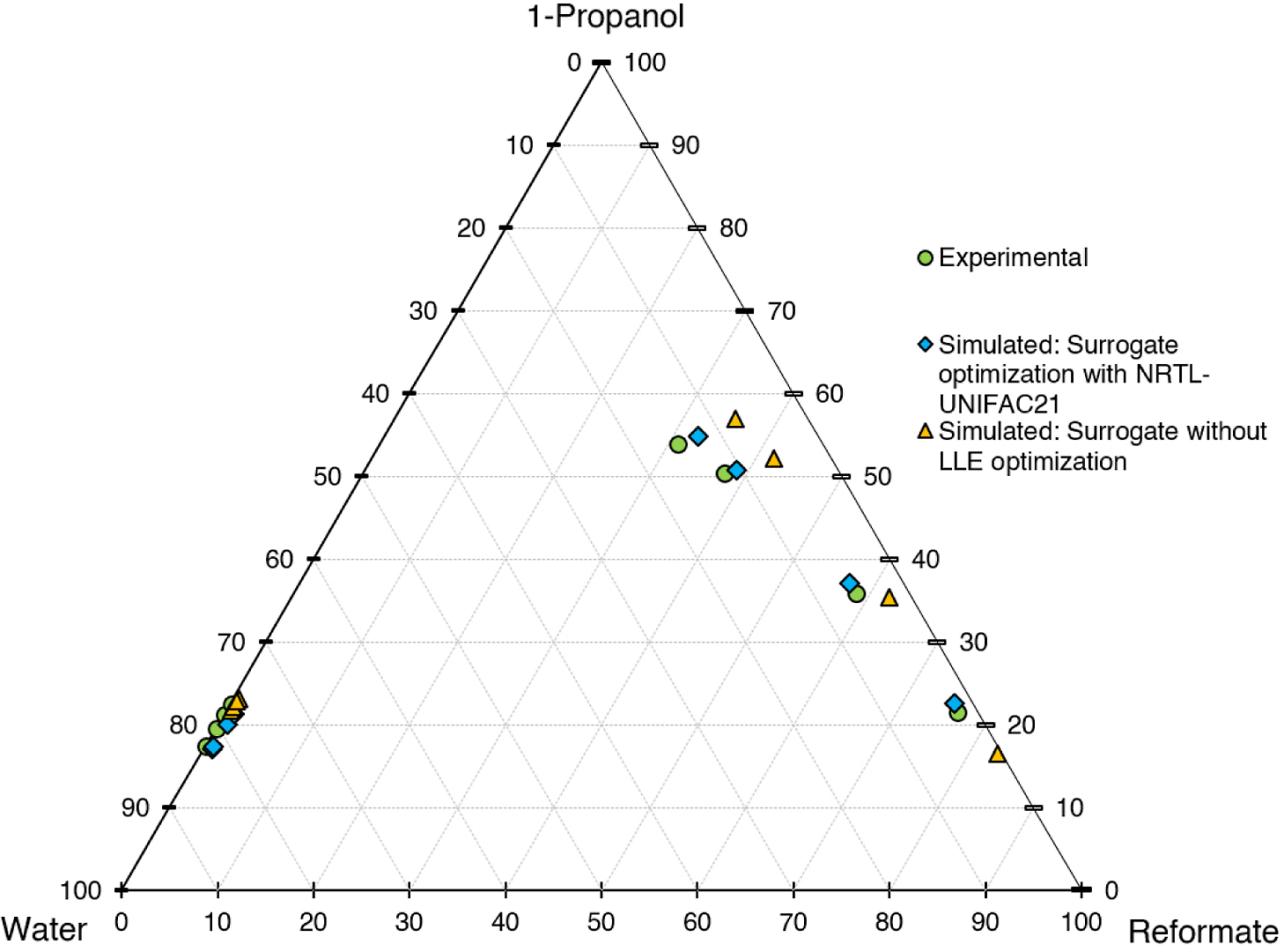
Ternary diagram of system 1-propanol–water–reformate, comparison between experimental LLE data of the validation set with simulated results with and without LLE-optimized surrogate, simulation conducted in AspenPlus V11.

As can be seen in Table 8, the LLE optimization comes at the slight expense of other target values. However, the deviations are still within an acceptable range. Furthermore, the focus of the LLE-optimized surrogate is the depiction of the liquid–liquid equilibrium, while other target values play a minor role. The NRTL-UNIFAC21 surrogate applied to the validation set yielded satisfactory results. The ternary diagram is shown in Figure 11, comparing the final performance of the LLE-optimized surrogate with the conventional one.

**Figure 12:**
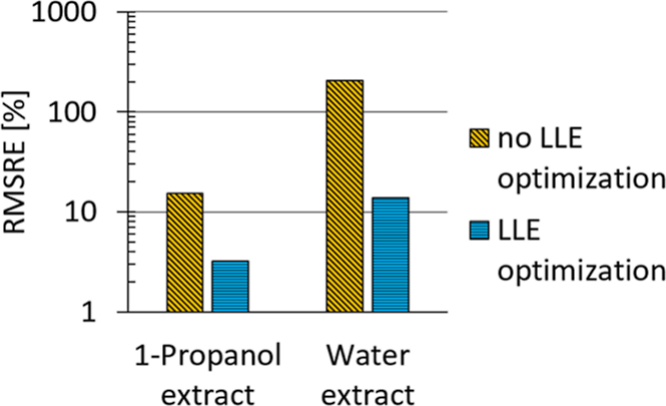
RMSRE for 1-propanol and water content in the extract for the validation set, comparison between LLE-optimized and conventional surrogates.

**Figure 13:**
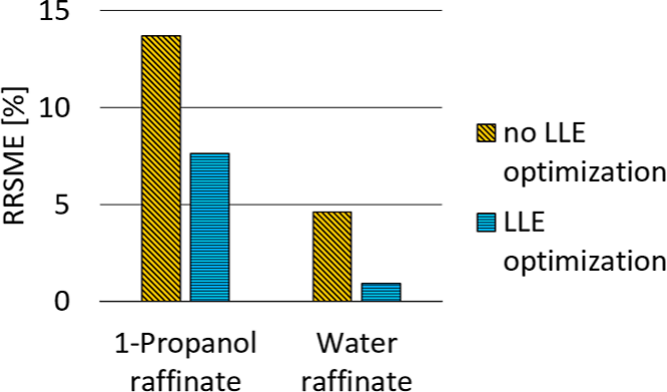
RMSRE for 1-propanol and water content in the raffinate for the validation set, comparison between LLE-optimized and conventional surrogates.

To get a quantitative statement about the surrogate performance, the root-mean-square relative errors (RMSRE) of both the conventional surrogate and the LLE-optimized surrogate are compared in Figures 12 and 13 for the validation set.

Significant improvements are achieved, with the error in water content prediction reduced by 93% and the error in 1-propanol prediction reduced by 79% for the extract phase. For the raffinate phase, the error in water content prediction is reduced by 80%, and the error in 1-propanol prediction is reduced by 44%.

### 4.4 Application to an Extraction Process

**Figure 14:**
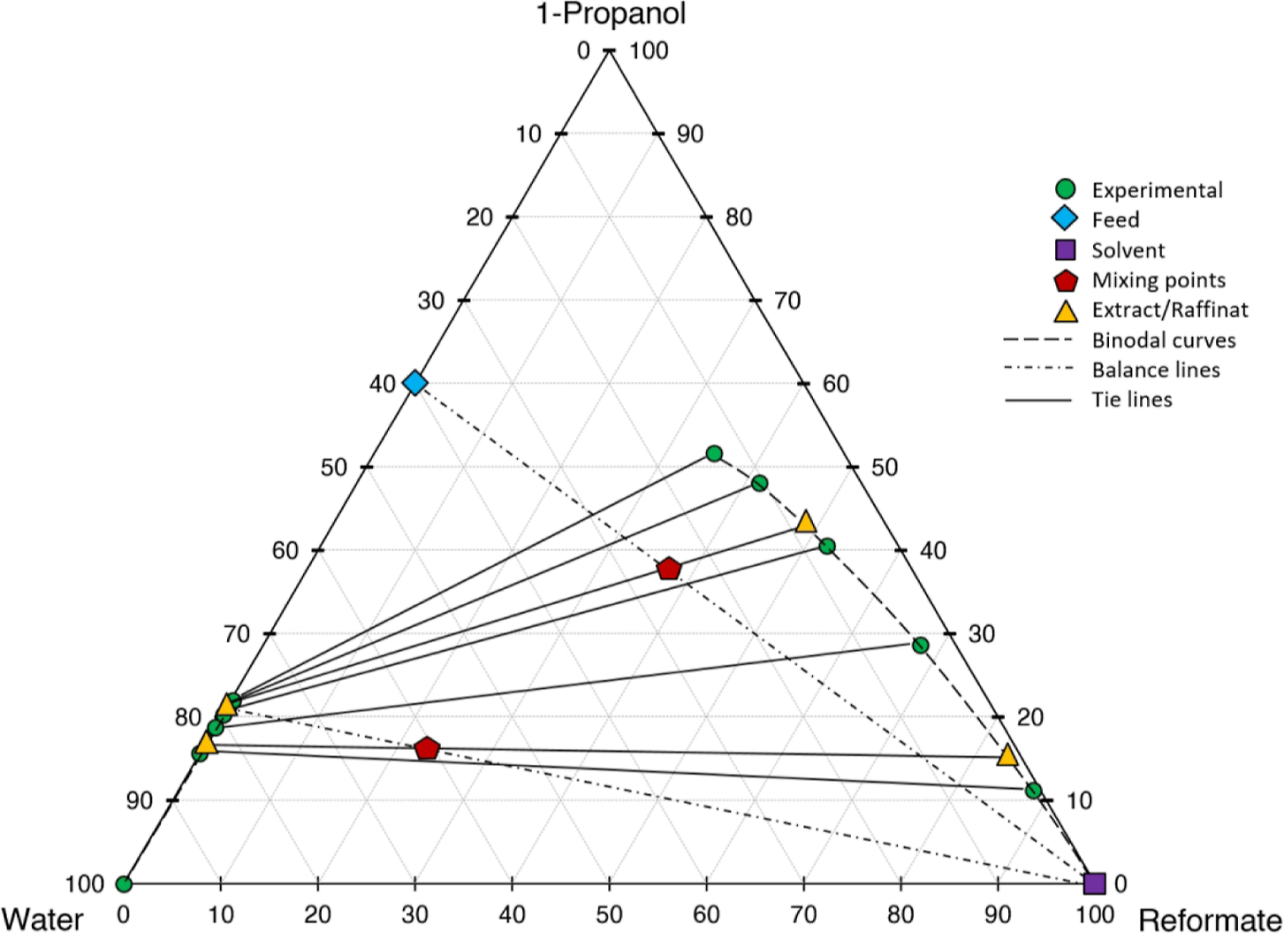
Stage construction for a two-stage cross-flow extraction process based on experimental LLE data; conjugation line for the construction of additional tie lines not shown in the diagram.

In the following, the practical benefit of the LLE-optimized surrogate shall be demonstrated by comparing the results of a two-stage cross-flow extraction process based on experimental LLE data with a simulated process in Aspen utilizing the LLE-optimized surrogate with NRTL-UNIFAC21. Figure 14 shows the stage construction for the two-stage cross-flow process based on experimental data.

**Figure 15:**
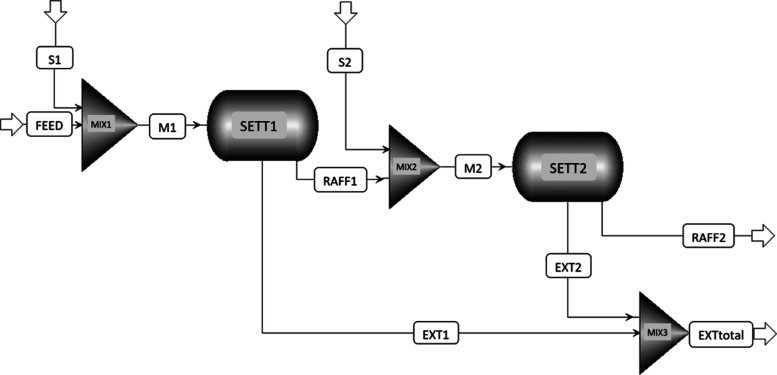
Flowsheet of the two-stage cross-flow process simulated in AspenPlus V11, S1: solvent stage 1, M1: mixing point 1, SETT1: settler stage 1, RAFF1: raffinate stage 1, EXT1: extract stage 1, S2: solvent stage 2, M2: mixing point 2, SETT2: settler stage 2, RAFF2: raffinate stage 2, EXT2: extract stage 2, and EXTtotal: combination of extracts from stages 1 and 2.

For each stage, fresh solvent is used. For the first stage, a solvent-to-feed (SF) ratio of 0.6 is used, and for the second stage, a SF ratio of 0.3 is used. The final product is obtained by combining the extracts from the first and second stage. The mixing point of the final extract (combining from stages 1 and 2) theoretically lies inside the 2-phase region. However, the amount of aqueous phase formed is negligibly small (0.6 kg of aqueous phase from 135 kg of mixture, according to Aspen) and is thus not considered in the calculation and comparison. The simulation was conducted in AspenPlus V11, with the flowsheet depicted in Figure 15. The results are shown in Table 9.

**Table 9:** Results for the Two-Stage Cross-flow Process: Comparison between Experimental Basis, Simulation with Conventional Surrogate, and Simulation with LLE-Optimized Surrogate for Streams EXTtotal and RAFF2

		surrogate	
extract	experimental	conventional	LLE optimized
1-propanol [wt %]	40.1	40.4	40.2
water [wt %]	7.6	3.5	7.1

		surrogate	
raffinate	experimental	conventional	LLE optimized
1-propanol [wt %]	16.6	19.5	15.4
water [wt %]	83.4	80.0	83.9

The process simulation with the LLE-optimized surrogate yields satisfactory results, with a significant improvement compared to the conventional surrogate achieved, especially for the water content in the extract. If the composition of the reformate solvent and thus its properties would change significantly between the extraction steps, a new surrogate would have to be calculated for the new composition and properties. However, the reformate composition does not change significantly during the course of the extraction since the reformate content in the raffinate is very low (<0.2 wt %). Therefore, the calculated surrogate is applicable for an arbitrary number of extraction stages and phase setups (cross-current vs counter-current).

## 5 Summary and Conclusions

In this work, a previously published methodology for the calculation of fuel surrogates was extended in view of the liquid–liquid extraction characteristics. This enables the thermodynamically rigorous simulation of extraction processes utilizing hydrocarbon fuels in commercial process simulators such as AspenPlus. First, LLE data for the ternary system 1-propanol–water–reformate were determined by means of shaking funnel experiments. The liquid–liquid distribution coefficients from these measurements were then incorporated into a surrogate calculation algorithm, which was expanded from an existing base algorithm for this application. With the obtained surrogates, the prediction accuracy of the LLE could be significantly improved compared to conventional surrogates without LLE optimization. Finally, a comparison was made for a two-stage cross-flow extraction process, one on the basis of experimental data and one simulated in Aspen with the LLE-optimized surrogate. This further confirmed the significant improvement in prediction accuracy as well as convenient applicability in commercial process simulators.

## Supporting Information Available

Analytical method details, surrogate compositions, and mass balance for the simulated extraction process (PDF)
