# Supplementary material for: Surrogates for Liquid–Liquid Extraction
Source: ACS Omega. 2023 Dec 14;8(51):49420–31. doi: 10.1021/acsomega.3c08140 (PMC10753540; doi:10.1021/acsomega.3c08140)
Supplement: Supplementary file 1 — ao3c08140_si_001.pdf [file ao3c08140_si_001.pdf]

Supporting Information

Surrogates for Liquid-Liquid Extraction

Maximilian Neubauer[a], Georg Lenk[b], Nikolai Josef Schubert[b], Susanne Lux[a], *Thomas Wallek[a]
[a]Graz University of Technology, Institute of Chemical Engineering and Environmental Technology, Inffeldgasse 25C, Graz, Austria
[b]OMV Downstream GmbH, Trabrennstraße 6-8, 1020 Vienna, Austria

*Corresponding author: Thomas Wallek: thomas.wallek@tugraz.at

Table S1: GC method

GC: Agilent 6890N
Column : Agilent J&W DB-624 UI 30*0.25*1.4
Solvent: DMSO
OVEN
Initial temp: 40 C (On) Maximum temp: 260 C
Initial time: 3.00 min Equilibration time: 0.01 min
Ramps:
Rate Final temp Final time
1 5.00 100 0.00
2 20.00 220 10.00
3 0 (Off)
Post temp: 0 C
Post time: 0.00 min
Run time: 31.00 min
FRONT INLET
Mode: Split
Initial temp: 250 C (On)
Pressure: 58 kPa (On)
Split ratio: 10:1
Split flow: 7.0 mL/min
Total flow: 10.3 mL/min
Gas saver: Off
Gas type: Nitrogen
COLUMN 1
Capillary Column
Model Number: 122-1334UI
Description: DB-624 UI
Max temperature: 260 C
Nominal length: 30.0 m
Nominal diameter: 250.00 um
Nominal film thickness: 1.40 um
Mode: constant flow
Initial flow: 0.7 mL/min
Nominal init pressure: 58 kPa
Average velocity: 20 cm/sec
Inlet: Front Inlet
Outlet: Front Detector
Outlet pressure: ambient
FRONT DETECTOR (FID)
Temperature: 280 C (On)
Hydrogen flow: 35.0 mL/min (On)
Air flow: 350.0 mL/min (On)
Mode: Constant column+makeup flow
Combined flow: 25.0 mL/min
Makeup flow: On
Makeup Gas Type: Nitrogen
Flame: On
Electrometer: On
Lit offset: 2.0

Table S2: Reference values for weighting the target criteria

target criterion	weighting factor
TBP	15
rho	0.01
RON	1
Molar mass	10
Aromatics fraction	0.1
x^E/x^R 1-Propanol	0.1
x^E/x^R Water	0.001

Table S3: Composition of conventional surrogate (TBP, density, RON, aromatics fraction)

Butane	2.08
Neopentane	1.25
Isopentane	1.73
Pentane	1.18
Neohexane	2.17
Isohexane	1.17
3-Methylpentane	0.98
Benzene	9.29
Isooctane	0.56
Methylcyclohexane	3.36
Toluene	15.43
Octane	0.49
Ethylcyclohexane	4.62
Ethylbenzene	10.10
p-Xylene	3.40
m-Xylene	2.31
o-Xylene	6.62
Isopropylbenzene	4.35
Propylbenzene	5.70
Mesitylene	8.99
1,2,4-Trimethylbenzene	4.01
Isobutylbenzene	0.16
1,2,3-Trimethylbenzene	2.57
Butylbenzene	1.36
trans-Decalin	0.91
cis-Decalin	1.28
Tetralin	2.59
Hexylbenzene	0.76
1-Methylnaphthalene	0.54

Table S4: Composition of surrogates with different g^E models for LLE flash algorithm

	no LLE optimization	UNIFACLL	NRTL-UNIFAC21	NRTL-UNIFACAspen	NRTL-UNIFACDMD	NRTL-UNIFACLL
Butane	2.08		15.46	22.31	32.83	
Neopentane	1.25					10.77
Isopentane	1.73					
Pentane	1.18					
Neohexane	2.17					
Isohexane	1.17					
3-Methylpentane	0.98					
Hexane	0.00					
Benzene	9.29	5.63	11.06	30.87	16.64	26.90
Isoheptane	0.00					
Heptane	0.00					
Isooctane	0.56	11.39				
Methylcyclohexane	3.36					
Toluene	15.43		28.02			5.39
Octane	0.49					
Ethylcyclohexane	4.62					
Ethylbenzene	10.10					
p-Xylene	3.40					
m-Xylene	2.31					
o-Xylene	6.62					
Nonane	0.00	4.63			7.38	
Isopropylbenzene	4.35					
Propylcyclohexane	0.00					
Propylbenzene	5.70					
Mesitylene	8.99					
2-Methyl-2-Phenylpropane	0.00	66.24				
1,2,4-Trimethylbenzene	4.01					
Isobutylbenzene	0.16					44.41
2-Phenylbutane	0.00					11.32
1,2,3-Trimethylbenzene	2.57	7.93				
Butylcyclohexane	0.00					
Butylbenzene	1.36					
trans-Decalin	0.91					
cis-Decalin	1.28					
Tetralin	2.59		45.45			
Hexylbenzene	0.76	3.63		2.98	5.22	
1-Methylnaphthalene	0.54					
Heptylbenzene	0.00	0.54		12.13		
Phenylbenzene	0.00			31.10	37.62	1.21
Octylbenzene	0.00			0.62	0.31	

Table S5: Mass balance from 2-stage cross-flow extraction process simulated in Aspen with conventional surrogate

no LLE optimization	Units	EXT1	EXT2	EXTtotal	FEED	M1	M2	RAFF1	RAFF2	S1	S2
Mass Flows	kg/hr	114.26	12.89	127.14	100.00	160.00	57.33	45.74	44.45	60.00	11.59
Mass Fractions											
Water		0.0385	0.0019	0.0348	0.4000	0.2500	0.6210	0.7784	0.8005	0.0000	0.0000
1-Propanol		0.4384	0.0957	0.4037	0.6000	0.3750	0.1728	0.2166	0.1952	0.0000	0.0000
Butane		0.0108	0.0186	0.0116	0	0.0078	0.0045	0.0004	0.0005	0.0208	0.0208
Neopentane		0.0065	0.0112	0.0069	0	0.0047	0.0028	0.0003	0.0003	0.0125	0.0125
Isopentane		0.0091	0.0156	0.0097	0	0.0065	0.0035	0.0000	0.0000	0.0173	0.0173
Pentane		0.0062	0.0107	0.0067	0	0.0044	0.0024	0.0000	0.0000	0.0118	0.0118
Neohexane		0.0114	0.0196	0.0122	0	0.0082	0.0044	0.0000	0.0000	0.0217	0.0217
Isohexane		0.0061	0.0105	0.0066	0	0.0044	0.0024	0.0000	0.0000	0.0117	0.0117
3-Methylpentane		0.0051	0.0088	0.0055	0	0.0037	0.0020	0.0000	0.0000	0.0098	0.0098
Hexane		0	0	0	0	0	0	0	0	0	0
Benzene		0.0485	0.0838	0.0521	0	0.0348	0.0193	0.0006	0.0006	0.0929	0.0929
Isoheptane		0	0	0	0	0	0	0	0	0	0
Heptane		0	0	0	0	0	0	0	0	0	0
Isooctane		0.0030	0.0051	0.0032	0	0.0021	0.0011	0.0000	0.0000	0.0056	0.0056
Methylcyclohexane		0.0176	0.0302	0.0189	0	0.0126	0.0068	0.0000	0.0000	0.0336	0.0336
Toluene		0.0808	0.1388	0.0867	0	0.0579	0.0316	0.0005	0.0006	0.1543	0.1543
Octane		0.0026	0.0044	0.0027	0	0.0018	0.0010	0.0000	0.0000	0.0049	0.0049
Ethylcyclohexane		0.0239	0.0423	0.0258	0	0.0173	0.0101	0.0010	0.0008	0.0462	0.0462
Ethylbenzene		0.0530	0.0910	0.0569	0	0.0379	0.0206	0.0002	0.0001	0.1010	0.1010
p-Xylene		0.0178	0.0307	0.0191	0	0.0128	0.0069	0.0001	0.0000	0.0340	0.0340
m-Xylene		0.0121	0.0208	0.0130	0	0.0087	0.0047	0.0000	0.0000	0.0231	0.0231
o-Xylene		0.0347	0.0597	0.0373	0	0.0248	0.0135	0.0001	0.0001	0.0662	0.0662
Nonane		0	0	0	0	0	0	0	0	0	0
Isopropylbenzene		0.0228	0.0392	0.0245	0	0.0163	0.0088	0.0000	0.0000	0.0435	0.0435
Propylcyclohexane		0	0	0	0	0	0	0	0	0	0
Propylbenzene		0.0299	0.0514	0.0321	0	0.0214	0.0116	0.0001	0.0001	0.0570	0.0570
Mesitylene		0.0472	0.0809	0.0506	0	0.0337	0.0182	0.0001	0.0000	0.0899	0.0899
2-Methyl-2-Phenylpropane		0	0	0	0	0	0	0	0	0	0
1,2,4-Trimethylbenzene		0.0210	0.0362	0.0226	0	0.0150	0.0082	0.0001	0.0000	0.0401	0.0401
Isobutylbenzene		0.0008	0.0015	0.0009	0	0.0006	0.0004	0.0000	0.0000	0.0016	0.0016
2-Phenylbutane		0	0	0	0	0	0	0	0	0	0
1,2,3-Trimethylbenzene		0.0135	0.0232	0.0145	0	0.0097	0.0052	0.0000	0.0000	0.0257	0.0257
Butylcyclohexane		0	0	0	0	0	0	0	0	0	0
Butylbenzene		0.0071	0.0123	0.0077	0	0.0051	0.0028	0.0000	0.0000	0.0136	0.0136
trans-Decalin		0.0047	0.0085	0.0051	0	0.0034	0.0021	0.0003	0.0002	0.0091	0.0091
cis-Decalin		0.0066	0.0119	0.0071	0	0.0048	0.0029	0.0004	0.0003	0.0128	0.0128
Tetralin		0.0134	0.0237	0.0144	0	0.0097	0.0056	0.0005	0.0003	0.0259	0.0259
Hexylbenzene		0.0039	0.0071	0.0042	0	0.0029	0.0017	0.0003	0.0002	0.0076	0.0076
1-Methylnaphthalene		0.0028	0.0049	0.0031	0	0.0020	0.0011	0.0000	0.0000	0.0054	0.0054
Heptylbenzene		0	0	0	0	0	0	0	0	0	0
Phenylbenzene		0	0	0	0	0	0	0	0	0	0
Octylbenzene		0	0	0	0	0	0	0	0	0	0

Table S6: Mass balance from 2-stage cross-flow extraction process simulated in Aspen with LLE optimized surrogate

NRTL-UNIFAC21	Units	EXT1	EXT2	EXTtotal	FEED	M1	M2	RAFF1	RAFF2	S1	S2
Mass Flows	kg/hr	122.26	13.05	135.31	100.00	160.00	49.33	37.74	36.28	60.00	11.59
Mass Fractions											
Water		0.0778	0.0039	0.0707	0.4000	0.2500	0.6180	0.8078	0.8389	0.0000	0.0000
1-Propanol		0.4343	0.1007	0.4022	0.6000	0.3750	0.1398	0.1828	0.1539	0.0000	0.0000
Butane		0.0751	0.1373	0.0811	0.0000	0.0580	0.0384	0.0027	0.0028	0.1546	0.1546
Neopentane		0	0	0	0	0	0	0	0	0	0
Isopentane		0	0	0	0	0	0	0	0	0	0
Pentane		0	0	0	0	0	0	0	0	0	0
Neohexane		0	0	0	0	0	0	0	0	0	0
Isohexane		0	0	0	0	0	0	0	0	0	0
3-Methylpentane		0	0	0	0	0	0	0	0	0	0
Hexane		0	0	0	0	0	0	0	0	0	0
Benzene		0.0540	0.0991	0.0584	0.0000	0.0415	0.0266	0.0008	0.0006	0.1106	0.1106
Isoheptane		0	0	0	0	0	0	0	0	0	0
Heptane		0	0	0	0	0	0	0	0	0	0
Isooctane		0	0	0	0	0	0	0	0	0	0
Methylcyclohexane		0	0	0	0	0	0	0	0	0	0
Toluene		0.1372	0.2497	0.1481	0.0000	0.1051	0.0666	0.0010	0.0007	0.2802	0.2802
Octane		0	0	0	0	0	0	0	0	0	0
Ethylcyclohexane		0	0	0	0	0	0	0	0	0	0
Ethylbenzene		0	0	0	0	0	0	0	0	0	0
p-Xylene		0	0	0	0	0	0	0	0	0	0
m-Xylene		0	0	0	0	0	0	0	0	0	0
o-Xylene		0	0	0	0	0	0	0	0	0	0
Nonane		0	0	0	0	0	0	0	0	0	0
Isopropylbenzene		0	0	0	0	0	0	0	0	0	0
Propylcyclohexane		0	0	0	0	0	0	0	0	0	0
Propylbenzene		0	0	0	0	0	0	0	0	0	0
Mesitylene		0	0	0	0	0	0	0	0	0	0
2-Methyl-2-Phenylpropane		0	0	0	0	0	0	0	0	0	0
1,2,4-Trimethylbenzene		0	0	0	0	0	0	0	0	0	0
Isobutylbenzene		0	0	0	0	0	0	0	0	0	0
2-Phenylbutane		0	0	0	0	0	0	0	0	0	0
1,2,3-Trimethylbenzene		0	0	0	0	0	0	0	0	0	0
Butylcyclohexane		0	0	0	0	0	0	0	0	0	0
Butylbenzene		0	0	0	0	0	0	0	0	0	0
trans-Decalin		0	0	0	0	0	0	0	0	0	0
cis-Decalin		0	0	0	0	0	0	0	0	0	0
Tetralin		0.2216	0.4093	0.2397	0.0000	0.1705	0.1105	0.0049	0.0031	0.4545	0.4545
Hexylbenzene		0	0	0	0	0	0	0	0	0	0
1-Methylnaphthalene		0	0	0	0	0	0	0	0	0	0
Heptylbenzene		0	0	0	0	0	0	0	0	0	0
Phenylbenzene		0	0	0	0	0	0	0	0	0	0
Octylbenzene		0	0	0	0	0	0	0	0	0	0
